# Influencing Factors of Phosphorus Mobility and Retention in the Sediment of Three Typical Plateau Lakes

**DOI:** 10.3390/toxics13020120

**Published:** 2025-02-03

**Authors:** Xue Wu, Yancai Wang, Lixin Jiao, Jia He, Hongbin Zhou, Zhengzheng Hao

**Affiliations:** 1 Faculty of Environmental Science and Engineering, Kunming University of Science and Technology, Kunming 650500, China; stellawu7@126.com; 2 Kunming Institute of Eco-Environmental Sciences, Kunming 650032, China; diamond_wyc@163.com (Y.W.); zhouh.b@163.com (H.Z.); 3 State Key Laboratory of Environmental Criteria and Risk Assessment, Chinese Research Academy of Environmental Sciences, Beijing 100012, China; 4 Yunnan Dianwei Environmental Protection Technology Co., Ltd., Kunming 650031, China; haozhengzheng2024@163.com

**Keywords:** lakes, sediments, phosphorus, retention, mobility

## Abstract

The mechanisms driving changes in the stability of phosphorus (P) in sediments under lake ecosystem degradation remain poorly understood. This study investigated the P-binding forms in sediments from three plateau lakes with different trophic states in Yunnan Province, China, aiming to elucidate the responses of sediment P compositions to human activities, lake trophic status, and dissolved organic matter (DOM) characteristics. The results showed that human activity directly contributed to sediment P retention. The trophic type of lake exerted a discernible effect on P mobility in the sediments, as eutrophic algae-type lakes had a higher content of sediment mobile-P. Moreover, the sediment DOM promoted the adsorption of BD-P and NH_4_Cl-P. Generally, exogenous pollution caused by human activity leads to lake eutrophication and a decline in lake ecosystem stability. This variation was largely influenced by water depth. A decrease in lake ecosystem stability leads to increased P mobility in sediments, which increases the risk of endogenous pollution. The DOM plays an important role in the mobility of sediment P. These insights offer a novel perspective for understanding how lake ecosystem characteristics are related to endogenous P loads in lakes.

## 1. Introduction

Phosphorus (P) is a crucial nutrient for aquatic vegetation growth; however, its excessive accumulation in water bodies leads to eutrophication, characterized by cyanobacterial blooms and the production of phycotoxins, posing severe ecotoxicological risks to aquatic ecosystems [1,2,3]. Lake sediments act as major reservoirs of P. Even when external P inputs are controlled, the release of internal P from sediments remains a key driver of lake eutrophication [4,5,6]. Sedimentary P exists in two main forms: mobile-P and immobile-P. Mobile-P includes weakly adsorbed phosphorus (NH_4_Cl-P), organic phosphorus (NaOH-nrP), and iron-bound phosphorus (BD-P), while immobile-P includes aluminum-bound phosphorus (NaOH-rP), calcium-bound phosphorus (HCl-P), and residual phosphorus (Res-P), each exhibiting distinct bioavailability and environmental behavior [7,8,9,10]. Among these, mobile-P is highly bioavailable and can readily support algal growth [11,12,13].

Dissolved organic matter (DOM), as the most reactive and bioavailable component of sedimentary organic matter (SOM), plays a pivotal role in lake eutrophication by influencing the geochemical cycling of P [14,15]. DOM includes various functional groups, such as carboxyl, hydroxyl, amino, and aromatic groups, that interact with P, as well as metal ions, to create complexes that affect the environmental behavior and bioavailability of P [16,17]. The interactions between DOM and P are variable and complex. For instance, the carboxyl groups in DOM can act as organic anions to partially replace phosphate adsorbed by the soil through competitive adsorption. Alternatively, soluble ternary complexes such as DOM-Fe-PO_4_ and DOM-Al-PO_4_ can be formed through complexation and dissolution, thereby enhancing P bioavailability [18,19]. Kurek et al. [20] highlighted the pivotal role of DOM in seasonal organophosphorus transformation. Humic-like components in DOM are recognized as key factors governing P adsorption and release [21]. Recent research has indicated that the distribution of the fluorescent components in DOM is closely associated with the adsorption characteristics of P in the sediments of Erhai Lake [22]. High levels of protein-like components can reduce P adsorption in sediments by decreasing the goethite content [22,23]. Furthermore, in Taihu Lake, DOM resulting from algal blooms under eutrophic conditions enhanced the reduction and dissolution of Fe and Mn oxides, and this process inevitably influenced the forms of Fe- and Mn-bound P in sediments, as well as the release of P [24].

Many researchers have studied P forms, DOM characteristics, and the binding mechanisms between P and DOM in lake sediments [25,26,27]. Zhao et al. [28] investigated how the composition and structural characteristics of organic matter affect the phosphorus forms in the sediments of Yangzonghai Lake. Chen et al. [29] investigated the effects of climate change and human activity on the P of the sediments in Dianchi Lake. Ni et al. [30] studied the effects of DOM on the degradation and release of diester P in the sediments of the low-nutrient Erhai Lake. However, many of these studies have been limited to individual lakes, with few reports on the binding forms of sediment P in lakes with different anthropogenic intensities and trophic states and their correlation with DOM. The origins of P and DOM in lake sediments encompass endogenous and exogenous sources, with external human activities and the hydrological, water quality, and ecological conditions of the lake, which play formidable roles in shaping the characteristics of DOM and its binding behavior with P [31,32]. This study investigated the forms of phosphorus in sediments from three plateau lakes in Yunnan Province, China: Dianchi Lake, Erhai Lake, and Yangzonghai Lake, each representing a different trophic state. It presents a comparative analysis of the basic characteristics of these lakes, the occurrence of sediment P forms, and the fluorescence characteristics of sediment DOM. The main objectives of this paper were to examine how DOM and human activities influence phosphorus retention in sediments and to explore how lake nutrient levels and ecological characteristics impact the mobility of phosphorus in lake sediments.

## 2. Materials and Methods

### 2.1. Overview of the Research Areas

Yunnan Province in China is home to Dianchi Lake, Erhai Lake, and Yangzonghai Lake, all of which display distinct variations in basin size, water depth, level of human activity, and trophic state.

Situated in the southern part of Kunming main city area, Dianchi Lake (N24°40′–25°02′, E102°36′–102°47′) is recognized as the sixth-largest freshwater lake in China. Dianchi Lake is separated into Caohai Lake and Waihai Lake by a dam, with a total basin area of 2920 km^2^ and a water area of 297.9 km^2^ [33]. Dianchi Lake is classified as an urban lake with a population of 5,453,500. The water quality of Dianchi Lake has deteriorated due to social and economic development since the late 1970s, leading to severe eutrophication and consistently low water quality below Grade V. However, after years of extensive treatment, the water quality was upgraded to Grade IV by 2018. However, it is still characterized as a typical eutrophic algal lake with a submerged vegetation coverage rate of approximately 3% [34]. Based on the 2018 investigation data on sediments in Dianchi Lake and the findings of Zhang et al. [35], the surface sediments of the lake are primarily black in color, with a foul odor, and the particle size is predominantly within the <250 μm range, with silt (4 to <63 μm) accounting for the largest proportion, averaging 60%, followed by clay (<4 μm), which constitutes an average of 19%.

Situated to the north of Dali City, Erhai Lake (N25°35′–25°58′, E100°5′–100°17′) is the seventh-largest freshwater lake in China. The lake covers a basin measuring 2565 km^2^, with a water surface area of 249.8 km^2^. This lake houses a basin population of 854,800 [36]. Since the 1990s, rapid economic development has caused a surge in the pollution load of the lake, leading to consistent elevation of nutrients in the lake. Consequently, the trophic status has gradually shifted from oligotrophic to mesotrophic. Currently, the water quality of Erhai Lake is categorized as Grades II–III, and the lake is in a transitional phase from mesotrophic to eutrophic. As a grass–algae mixed lake, it houses relatively abundant aquatic vegetation, with a vegetation coverage of approximately 10%. Although large-scale cyanobacterial blooms occurred in 1996, 2003, and 2013, the water quality of this lake has remained satisfactory [37]. The sediments of Erhai Lake appear brown, exhibiting a foul odor. The sediment primarily comprises clay, silt, fine sand, and coarse sand. The clay particles range from 0.57 to 1.1 μm, with a percentage content varying between 2.6% and 12.2%, and the fine silt particles range from 1.5–6.4 μm, with a percentage content ranging from 7.5% to 89.8% [38].

Situated in the southeastern part of Kunming, Yangzonghai Lake (N24°51′–24°58′, E102°58′–103°01′) spans 286 km^2^ in basin area and 31.3 km^2^ in water area. The lake is a typical deep-water lake with a population of 61,400. Yangzonghai Lake was considered oligotrophic before the 1990s; however, the introduction of cage fish culture in the late 1990s accelerated the decline in water quality. Moreover, the period from 1996 to 1998 witnessed multiple occurrences of cyanobacterial blooms. In 2008, the lake experienced serious As contamination stemming from industrial operations. The water depth of the lake makes the aquatic vegetation community relatively simple, mainly distributed in the shallow water area of the lake bank. Presently, this lake is categorized as a mesotrophic algae lake [39]. The surface sediments of Yangzonghai Lake are brown and have a slight odor. They consist primarily of clay, fine silt, and sand. The clay particles range from 0.57 to 1.1 μm, accounting for 2.6% to 12.2% of the sediment, and the fine silt particles range from 1.5 to 6.4 μm, comprising 7.5% to 89.8% of the total sediment [38].

More information on the basic characteristics of the three lakes can be found in Section S1 of the Supplementary Material.

### 2.2. Sampling Site Layout

**Figure 1:**
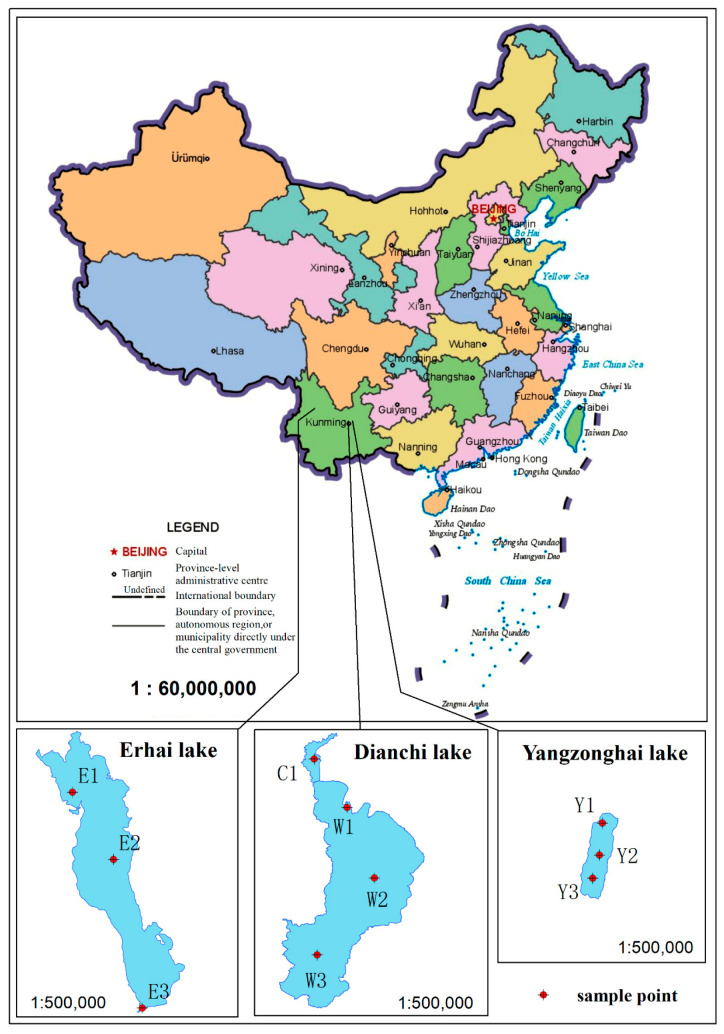
Layout of sediment sampling sites.

Ten sediment sampling sites were set up in the Dianchi, Erhai, and Yangzonghai lakes. Among them, four monitoring sites were set up in Dianchi Lake, located in Caohai Lake, as well as the northern, middle, and southern regions of Waihai Lake, which were numbered C1, W1, W2, and W3, respectively. Similarly, three monitoring sites were set up in Erhai Lake, located in the northern, central, and southern parts of Erhai Lake, denoted as E1, E2, and E3, respectively. Three monitoring sites were set up in Yangzonghai Lake, located in the north, center, and south of the lake and labelled Y1, Y2, and Y3, respectively. The specific sampling site positions are illustrated in Figure 1.

### 2.3. Sampling and Analysis

#### 2.3.1. Sediment Sampling

In March 2021, surficial sediment samples from 10 monitoring sites were collected using a mud bucket at a depth of 0–10 cm. The samples were carefully sealed in airtight plastic bags and placed in refrigerated storage before being transported to the laboratory for natural air drying. After air drying, surplus organic and inorganic residues were removed from the sediment samples, which were subsequently pulverized into a fine powder using a sieve for future analysis [25].

#### 2.3.2. Analysis of SOM and DOM

The SOM content of 10 sediment samples collected from Dianchi Lake, Erhai Lake, and Yangzonghai Lake was determined using the weight difference method [40]. After adjusting the moisture content, 40 g of the treated sediment was combined with deionized water in a 1:5 ratio. The blend was agitated at 300 rpm for a full day, followed by centrifugation at 4000 rpm for 10 min. The supernatant was filtered through a 0.45 μm glass fiber filter (pre-fired at 450 °C and maintained at a constant temperature for 5 h). The filtered solution, referred to as DOM solution for the experiment, was stored in the dark at 4 °C [41].

Three-dimensional fluorescence spectra of the DOM samples were analyzed. The excitation wavelength range was 200–450 nm, with a scanning interval of 5 nm. With a similar scanning interval, the emission wavelength range was set to 250–600 nm. The slits of the excitation and emission units were adjusted to 10 nm, the photomultiplier tube was operated at 400 V, and the scanning speed was settled to 2400 nm/min.

Fluorescence data were analyzed using the PARAFAC method to identify the DOM fluorescence components and their intensities. The data were imported into 3D specTALYZE 1.5 for noise removal and scattering correction. A three-dimensional projection or contour map was created based on the excitation and emission wavelengths and intensities. The specific excitation and emission spectra of each component (C1, C2, C3, and C4) were compared with the OpenFluor database to identify the corresponding substances.

#### 2.3.3. Determination and Analysis of P

The sediment sample weighing 0.2 g underwent combustion at 450 °C for 3 h and was then transferred into a 50 mL centrifugal tube for centrifugation. The total phosphorus (TP) was quantified using standard methods of alkaline K persulfate digestion and ultraviolet spectrophotometry [42]. The sediment P fractions were extracted using the continuous extraction method [9,43]. In the extraction process, 1 g of sediment samples were first weighed. Then, NH_4_Cl-P, BD-P, NaOH-rP, HCl-P, and Res-P were extracted with NH_4_Cl (1 M), Na_2_S_2_O_4_/NaHCO_3_, NaOH (0.1 M), HCl (0.5 M), and NaOH (1 M) reagents, respectively. After extraction, the above extraction solution was centrifugated for 10 min at 4000 r/min and filtered through a 0.45 um filter membrane. The content of phosphorus in the extraction solution was determined using molybdenum–antimonic spectrophotometry. NaOH-nrP was obtained by subtracting the above inorganic phosphorus from the TP.

#### 2.3.4. Water Quality Analysis

The water quality monitoring parameters in this study consisted of chemical oxygen demand (COD), TP, ammonia nitrogen (NH_3_-N), total nitrogen (TN), chlorophyll a (Chla), and the trophic state index. The preservation and monitoring techniques used for the samples were based on the guidelines provided in the 4th Edition of Monitoring and Analysis Methods for Water and Wastewater [44].

#### 2.3.5. Data Analysis and Quality Assurance

We analyzed the correlations between sediment phosphorus forms and both sediment DOM characteristics and human activity indicators in the watershed. The Pearson correlation coefficient was used to examine the relationships between these variables in the three lakes. The sediment indicators included TP content, phosphorus forms, DOM content, SOM mass fraction, and fluorescence intensity of the four components. The human activity indicators included GDP per unit area, population density, urbanization rate (urban population/total population), and the proportion of urban area (urban land area/total land area). A Pearson correlation analysis was conducted, and correlation matrix heatmaps were generated. Significant correlations were considered with *p*-values < 0.05.

In this paper, graphs were created using ArcGIS 10.8 and Origin 2021. To ensure data reliability and accuracy, 3 samples were collected at each site, mixed for representativeness, and promptly transported to the lab. The samples were sieved through a 100-mesh sieve for uniformity, and repeated experiments and blank tests were conducted. All reagents used were of analytical grade or higher, meeting industry standards and being used before their expiration dates.

## 3. Results

### 3.1. Basic Features of the Three Typical Lakes

Among the three typical plateau lakes investigated in this study, Dianchi Lake exhibited moderate pollution, with water quality classified as Class IV, while Erhai Lake and Yangzonghai Lake had relatively good water quality, both classified as Class II. The chlorophyll a (Chla) concentrations in Dianchi Lake were 10.66 and 11.88 times greater than those in Erhai Lake and Yangzonghai Lake, respectively. Similarly, the trophic level index was 1.58 times higher than in Erhai Lake and 1.51 times higher than in Yangzonghai Lake. The TP concentration of Dianchi Lake water was about 0.06 mg/L, and those of Erhai Lake and Yangzonghai Lake were both about 0.02 mg/L.

Analysis of the sediments from the three lakes indicated that the average contents of SOM and DOM in Dianchi Lake were 19.49% and 1640.63 mg/kg, respectively, which were the highest among the three lakes. Both SOM and DOM peaked in the Caohai region, significantly surpassing other areas. In contrast, Erhai Lake and Yangzonghai Lake had only 24% and 32% of the SOM content of Dianchi Lake, respectively, with their DOM contents at approximately 62% and 81% of Dianchi Lake’s levels. Spatial variation in SOM and DOM distribution was relatively low in Erhai and Yangzonghai Lakes. The sediment DOM of Erhai Lake was mainly concentrated in the north and south and was relatively low in the middle area. The sediment DOM of Yangzonghai Lake was the highest in the middle, second-highest in the south, and lowest in the north.

Dianchi Lake also exhibited significantly higher porewater SRP concentrations compared to Erhai Lake and Yangzonghai Lake: 7.91 and 6.06 times higher, respectively. The SRP concentrations were highest in the Caohai region and the northern part of Waihai, while the middle and southern parts of Waihai showed relatively low concentrations. In Erhai Lake, the SRP levels were higher in the middle and southern regions compared to the northern part, where sediment acted in an “adsorption” state, with SRP concentrations lower than those in the overlying water. In Yangzonghai Lake, the SRP concentrations were highest in the middle, followed by the north and lowest in the south.

Detailed information can be found in Section S2 of the Supplementary Material.

### 3.2. Characteristics of P-Binding Forms in Sediments

**Figure 2:**
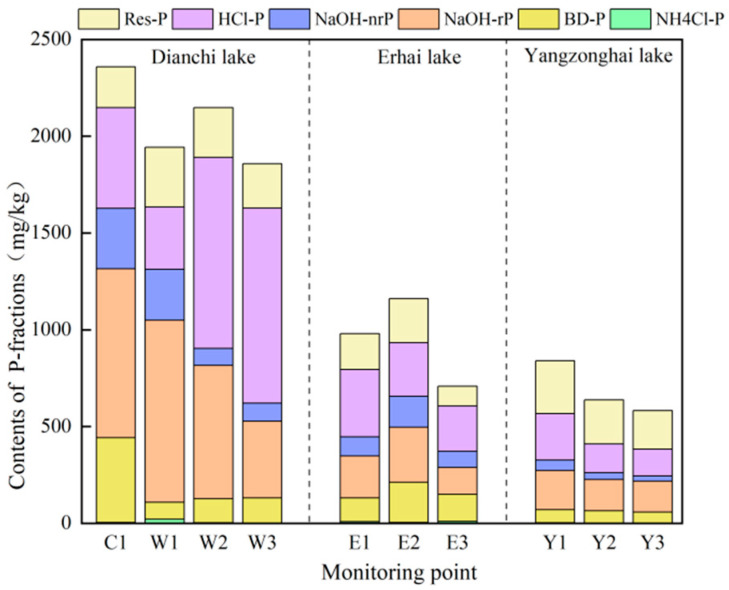
P form contents in the sediments of various monitoring sites in the lakes.

The average sediment TP contents in the Dianchi, Erhai, and Yangzonghai lakes were 2395.24, 1017.49, and 896.64 mg/kg, respectively. Dianchi Lake has a relatively high TP content, approximately twice that of Yangzonghai and Erhai Lakes. Different P-binding forms in the sediments demonstrate variations in P mobility and bioavailability [45]. Figure 2 shows the contents of different forms of P in the sediments of the Dianchi, Erhai, and Yangzonghai lakes. In Caohai Lake, the predominant form of P was NaOH-rP, which accounted for 34.72% of the total P content. Conversely, HCl-P was the featured P form in the Waihai and Erhai lakes, accounting for 33.10% and 28.70%, respectively, on average. In particular, the HCl-P in Waihai Lake was mainly distributed in the central and southern regions, whereas the other P forms were principally distributed in the northern region. HCl-P in Erhai Lake prevails extensively in the northern region, whereas other forms are concentrated in the central region. Res-P emerged as the leading form of P in Yangzonghai Lake, accounting for 25.99% of the P content. Regardless of form, the P content gradually decreased from north to south in Yangzonghai Lake. NH_4_Cl-P is the least concentrated form of P in lake sediments, comprising <1% of the TP. Being highly biologically active, NH_4_Cl-P is readily released into water, absorbed, and used by aquatic plants, resulting in a low content in the sediment [46].

### 3.3. DOM Fluorescence Characteristics of Sediments

**Figure 3:**
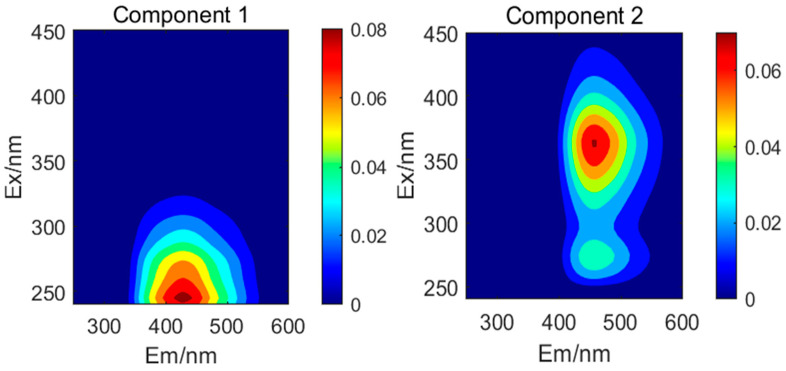

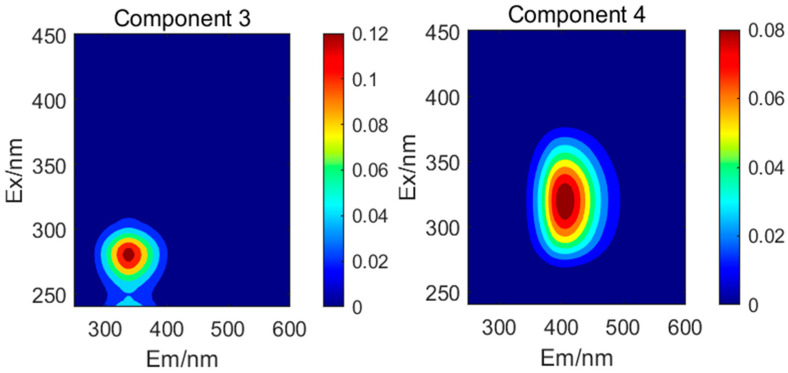
Fluorescence components of sediment DOM.

The DOM contents in the sediments of Dianchi Lake, Erhai Lake, and Yangzonghai Lake were analyzed based on their three-dimensional fluorescence spectrum. Details of the fluorescent components of sediment DOM in the three lakes are provided in Figure 3. Four fluorescent components were identified based on the DOM fluorescence characteristics of the lake sediments. Component C1 (Ex: 245 nm, Em: 430 nm) corresponded to a terrestrial humic-like substance [47]. Component C2 (Ex: 275/365 nm, Em: 455 nm) coincides with a fulvic acid-like substance known for its stability and resemblance to humus [48]. Components C3 (Ex: 280 nm, Em: 335 nm) and C4 (Ex: 320 nm, Em: 405 nm) conformed to protein tryptophan components and terrestrial humic acids, respectively [49,50]. Overall, C1, C2, and C4 are humic-like substances, whereas C3 is protein-like.

**Figure 4:**
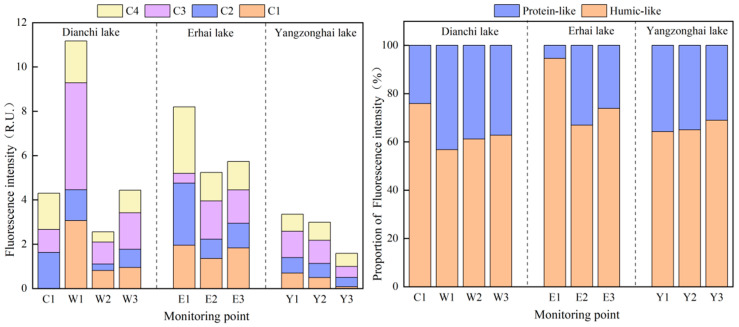
Fluorescence intensities and relative proportions of sediment DOM at each monitoring site.

The fluorescence intensities and relative proportions of sediment DOM from various monitoring sites in the lake are shown in Figure 4. Erhai Lake exhibited the highest total average fluorescence intensity, followed by Dianchi Lake, with Yangzonghai Lake displaying the lowest average. It is worth noting that the northern region of Dianchi Lake exhibited a significantly higher total fluorescence intensity compared to the other areas of the lake. Moreover, the northern zone of Erhai Lake showed higher total fluorescence intensity than the southern and central zones. The total fluorescence intensity in Yangzonghai Lake decreased from the northern to the southern zones. The average proportions of the sum of C1, C2, and C4 in the sediments of Dianchi, Erhai, and Yangzonghai were 68.06%, 78.47%, and 66.08%, respectively, indicating that the sediment DOM in the three lakes mainly comprised humic-like substances. Humic-like components were more abundant in Caohai Lake compared to Waihai Lake, while in Waihai Lake, the concentration of humic-like substances displayed a declining gradient from south to north. In Erhai Lake, the northern region exhibited a significantly higher concentration of humic-like components compared to the central and southern regions. Additionally, a rise in humic-like material levels from north to south was confirmed in Yangzonghai Lake.

### 3.4. Correlations Between Sediment P Fractions, DOM, and Human Activities

**Figure 5:**
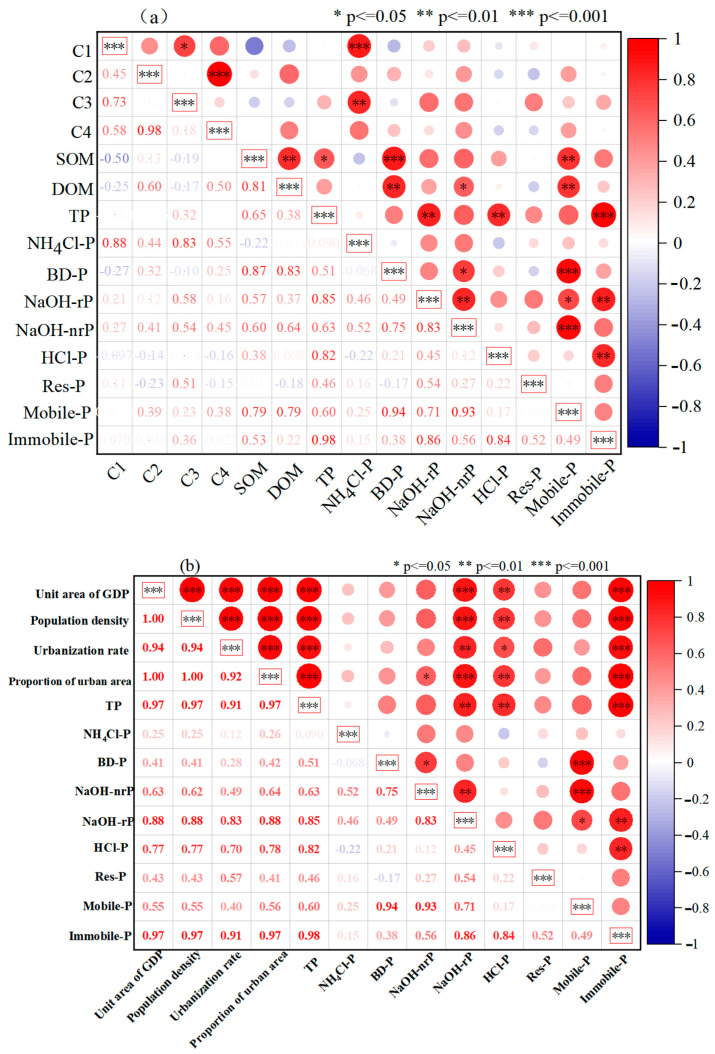
Correlation analysis of DOM and human activities with phosphorus. (**a**) The correlation coefficients between the P fractions and DOM characteristics. (**b**) The correlation coefficients between the P fractions and human activity indicators.

This study analyzed the Pearson correlation of DOM and human activities with sediment P fractions. As shown in Figure 5a, the DOM content was positively correlated with mobile-P (*p* < 0.01, *n* = 10), BD-P (*p* < 0.01, *n* = 10), and NaOH-nrP (*p* < 0.05, *n* = 10) in the sediments. A strong positive relationship was observed between the NH_4_Cl-P and the terrestrial humic-like substance C1 component (*p* < 0.001, *n* = 10) and protein-like C3 component (*p* < 0.01, *n* = 10). As illustrated in Figure 5b, the indicators of human activity intensity were significantly and positively correlated with TP (*p* < 0.001, *n* = 10), immobile-P (*p* < 0.001, *n* = 10), NaOH-rP (*p* < 0.01, *n* = 10), and HCl-P (*p* < 0.05, *n* = 10) in the lake sediments.

## 4. Discussion

### 4.1. The Influence of Sediment DOM on P Mobility

Despite its relatively low content in sediments, DOM plays a crucial role in influencing the potential mobility of phosphorus within sediments [51]. A significant positive correlation was found between the DOM and mobile-P contents in the sediments. Mobile-P, with its relatively short cycling timescale of approximately 10 years, is considered a critical contributor to lake eutrophication [13,52]. Among the various P-binding forms, DOM mainly influences the adsorption of BD-P in sediments and impacts the retention of NaOH-nrP.

In sediments, BD-P primarily consists of phosphorus bound to Fe oxides, with a smaller fraction associated with Mn oxides, and its release is predominantly controlled by redox conditions at the sediment–water interface [52]. In an anaerobic water environment, BD-P is released in high amounts from sediments and significantly affects the P concentration in the water. Previous studies indicated that BD-P is more likely to be retained in sediments in regions affected by eutrophication [53]. Therefore, BD-P often serves as a key indicator of endogenous P load in lake eutrophication scenarios [54,55]. This study showed that BD-P was the leading component of mobile-P in the sediments (53% on average), with a significant positive correlation observed between BD-P and the DOM content in the sediments. The significance of DOM in sediments is underscored, as it increases the potential for lake eutrophication by altering the interaction between Fe oxide and P in sediments. The BD-P content of the sediments was closely linked to the binding forms of Fe oxide. Typically, amorphous Fe oxide has a larger surface area and greater P adsorption capacity than crystalline Fe oxide [56]. Studies have shown that low-molecular-weight organic compounds produced via organic matter decomposition can form organic–metal chelates with stable chemical structures by bonding with metals, thereby augmenting P adsorption capacity [57].

NaOH-nrP primarily originates from the deposition of biological organisms [52,58,59]. A significant positive correlation was observed between the content of DOM and NaOH-nrP in the sediments. DOM is an important P reservoir in sediments; additionally, it may be related to the mechanisms of microbial action in the organophosphate cycle. Recent studies have found that the DOM humification process is conducive to the conversion of certain diester P species into RNA mononucleotide and β-glycerophosphoric acid, and inositol hexaphosphate can readily bind with polymer humus to form complex organophosphorus [25]. In addition, some studies have identified a significant negative correlation between the NaOH-nrP content and O/C and (O + N)/C [28]. In general, the higher ratios of O/C and (O + N)/C suggest that an increased number of functional groups, such as carbonyl, hydroxyl, carboxyl, amino, and nitro groups, are present in the aromatic core of DOM. Therefore, the humification process of DOM is likely to significantly affect the transformation and retention of organic P, and the specific underlying mechanisms warrant further investigation.

A strong positive correlation was found between the NH_4_Cl-P content in the sediments and the terrestrial humic-like C1 component, as well as the protein-like C3 component. The adsorption of NH_4_Cl-P in sediments is greatly influenced by the composition and structure of DOM. As NH_4_Cl-P is weakly adsorbed in sediments that comprise P in interstitial water, P binds to CaCO_3_ and separates from the degradation of bacterial cells in deposited plant debris [60,61]. The hydrophilic groups of the DOM compete with phosphate for adsorption or anion substitution, thereby inhibiting phosphate adsorption [62,63]. Previous studies have indicated that as the degree of humification of DOM increases, so does its molecular weight, whereas the number of functional groups such as hydroxyl, carboxyl, and carbonyl groups on aromatic nuclei decreases, creating a more conducive environment for the adsorption of NH_4_Cl-P in sediments [64,65,66]. In addition, the protein-like component of DOM serves as the primary carrier of soluble organophosphorus. Microbial degradation of soluble organophosphorus directly influences P adsorption in interstitial water and sediment through a microbial regulation mechanism [67], subsequently modulating the NH_4_Cl-P content in the sediments.

### 4.2. Human Activities Leading to P Retention in the Lake Sediments

Relevant studies have shown that human activities, including sewage discharge, land utilization, agricultural production, and phosphate mining, cause significant P pollution in sediments [68,69,70]. This study found that human activity intensity was significantly and positively correlated with TP, immobile-P, NaOH-rP, and HCl-P in the lake sediments.

This study revealed a direct correlation between the level of human activity in the lake basin and the TP concentration in the sediment, with higher intensity leading to increased sediment TP content. Higher population density and urbanization result in an elevated exogenous pollution load. As sinks for exogenous pollution, lake sediments inevitably accept substantial exogenous P loads, thereby augmenting the TP content of sediments. Among the three lakes in this study, Dianchi Lake had the highest sediment TP content, which was affected by the pollution intensity and pollution source characteristics (Section S3 in the Supplementary Material).

The level of human impact on lakes is positively correlated with the amount of immobile-P retained in lake sediments [71,72]. However, the intensity of human activities does not correlate with mobile-P because it displays strong mobility and bioavailability and is easily transformed [73]. Sediment immobile-P in the Dianchi, Erhai, and Yangzonghai lakes was mainly distributed in areas with marked human and agricultural activities. This phenomenon likely corresponded to the notable presence of NaOH-rP and HCl-P, which are easily influenced by human and agricultural activities [74,75].

A strong positive relationship existed between the proxy index measuring human activity intensity and NaOH-rP and HCl-P concentrations. NaOH-rP, an indicator of human activity, can be distinguished from industrial wastewater and domestic sewage [8]. Typically, P resides in urban lake sediments in the NaOH-rP form [76]. The NaOH-rP contents in Caohai Lake and Northern Waihai Lake were relatively high. After the 1960s, owing to the advancement of industrialization and urbanization, large portions of land in Caohai and the northern region of Waihai Lake in the Dianchi Lake Basin developed heavily, leading to an increase in population density [77]. Large volumes of industrial wastewater and domestic sewage are released into the lake, and NaOH-rP has been identified as a stable P form that tends to accumulate in sediments [78,79]. Compared with Dianchi Lake, the proportion of NaOH-rP in Erhai Lake and Yangzonghai Lake was relatively low, as they are categorized as non-urban lakes with a relatively lower population density, thus experiencing less impact from human activities [80,81]. The NaOH-rP in Erhai Lake was primarily distributed in the central zone. This is because the central zone is adjacent to Wase Town, where human activities and the introduction of Fe and Al have affected the region [82]. The spatial distribution of NaOH-rP in Yangzonghai Lake demonstrates a decreasing trend from the northern to the southern zone, likely because of the relatively high intensity of human activity in the northern zone of Yangzonghai. Domestic sewage is discharged into the lakes through the Baiyi River on the northern bank [83].

In contrast to NaOH-rP, HCl-P was widespread in the central and southern zones of Dianchi Lake. This was because the southern zone of Dianchi Lake is a geologically P-rich area with intensive mining and agricultural activities. Phosphorite extraction and fertilizer use have resulted in a significant influx of P into Dianchi Lake via runoff and erosion, eventually depositing into sediments [84]. HCl-P is the major P form in Erhai Lake, primarily distributed in the northern zone of Erhai Lake. The Miju River in the north contributes 51% of the water in Erhai Lake. Eryuan County, through which the Miju River flows, has a high population density and is mainly known for its agricultural practices, which result in significant agricultural nonpoint-source pollution. Agricultural runoff is the main source of P in Erhai Lake, with agricultural activities leading to increased HCl-P levels in sediments in the northern area of the lake [74]. Zhang et al. [56] showed that in lakes dominated by agriculture, the P forms in sediments are mainly HCl-P. The percentage of HCl-P in Yangzonghai Lake was lower than that of the Dianchi and Erhai lakes, indicating a decline from the northern region to the southern region. This is because the population and agriculture of Yangzonghai Lake are concentrated in the northern zone, facilitating the generation of HCl-P deposits.

### 4.3. The Impact of Lake Trophic and Ecological Types on Sediment P Mobility

Lakes in China are categorized based on the nutrients in the lake water, quantity of living biota, and primary producer species [85]. Currently, the coverage of aquatic vegetation in Dianchi Lake is <5%, and the algal biomass reaches the order of 10^7^–10^8^. This lake is a typical algal-type eutrophic shallow lake with an average depth of 4.4 m [44,61,86]. The coverage of aquatic vegetation in Erhai Lake is 13.2%, and the algal biomass reaches 10^7^ orders of magnitude. Currently undergoing a delicate transition from grass-dominated to algae-dominated, the lake is a typical grass–algae mixed type with a medium nutrient level and an average depth of 10.5 m [22,61,87]. Yangzonghai Lake has moderate nutrient levels and a typical depth of 20.13 m [88]. Its aquatic vegetation coverage is low, and the algal biomass is approximately 10^6^. The three lakes examined in this study exhibited high heterogeneity in terms of nutrient levels, aquatic biodiversity, richness, and water depth. This inevitably affects the retention and mobility of P in sediments.

Dianchi Lake exhibited the highest mobile-P content in its sediments, likely influenced by the nutrient levels within the lake. Organic materials are abundant in the sediments of eutrophic lakes. On the one hand, DOM is a P reservoir in sediments [89]. On the other hand, humus in DOM can form organic and inorganic complexes with Fe and Al, providing important adsorption sites for inorganic P and promoting P adsorption in sediments [90]. BD-P was the main component of mobile-P, indicating that high sediment DOM content was conducive to the adsorption of mobile-P. Liu et al. [41] studied Taihu Lake and showed that the biogeochemical cycles of P and OM are significantly influenced by Fe. The OM produced from macrophyte-dominated lakes has better aromatic properties, and Fe preferentially combines with highly aromatic compounds to form more OM-Fe-P and promotes the adsorption of P. Bao et al. [91] studied Chaohu Lake and noted that humic-like components could affect the dynamics of internal P through the formation of organic colloids and organic–inorganic ligands.

Erhai Lake and Yangzonghai Lake are mesotrophic lakes; however, Erhai Lake has a higher content and proportion of mobile-P in its sediments. Aquatic plants in lakes can affect the binding form of P in sediments by influencing its absorption and release [92]. Wang et al. [93] reported that synergistic interactions between aquatic plants and Fe-oxidizing bacteria can enhance BD-P formation and fixation in the sediment.

Water depth also has a significant influence on the form of P in the sediments. With increasing lake depth, there is a decrease in the presence of mobile-P in the sediment, indicating enhanced mobility of P in shallow lakes or the shallow parts of lakes. The richness and diversity of aquatic organisms are closely related to water depth, and shallow lakes or areas typically maintain greater biodiversity and richness. A recent study found that water depth influences phosphorus retention in sediments, primarily through its role in regulating ecosystem transitions [61]. Previous investigations have found a notable decline in P retention efficiency in sediments after a transition from a grass-dominated ecosystem to one dominated by algae [94]. In contrast, the potential mobile-P load of the sediment was significantly increased [52].

Specifically, the sampling point (C1) in Caohai Lake of Dianchi had the minimum water depth and maximum mobile-P content, which was 2–9 times higher than the mobile-P content of other sediment samples. The sampling point in the northern part of Waihai Lake (W2) had a mobile-P content 1.7–4.4 times higher than that of other sediment samples. This disparity was attributed to cyanobacterial accumulation in the northern zone of Dianchi Lake, which was influenced by exogenous inputs from the primary urban area of Kunming in the northern and southeastern wind directions. Algal blooms lead to anaerobic conditions and the release of BD-P from the sediment. The breakdown of algae and the deposition of dead algal debris may result in the build-up of biogenic organic P, thereby improving the mobility of sediment P [95]. In contrast, the southern zone of Waihai Lake features a grassy lake with submerged vegetation historically, which may promote the transformation of mobile-P in the sediments into immobile-P [96]. Therefore, the mobile-P in the southern zone of Waihai Lake was relatively low.

Moreover, submerged vegetation was distributed in the northern and southern areas of Erhai Lake, whereas the central zone lacked vegetation because of water depth [97,98]. Mobile-P levels in Erhai Lake reached their highest at the center, whereas deeper lake depths hindered the mineralization and decomposition of NaOH-nrP. The central area of Erhai Lake had the smallest proportion of HCl-P compared with the other two sites because of the lack of submerged plants that helped accumulate HCl-P [99]. The central zone of Erhai Lake exhibited a notable depth with scarce submerged plant growth.

Yangzonghai Lake features Res-P as the primary form of P, which is mainly composed of macromolecular organophosphorus or other insoluble P [100]. Compared with eutrophic lakes, organic detrital P in oligotrophic lakes is easily transformed into insoluble P [101]. Compared to Dianchi Lake and Erhai Lake, the algae and aquatic plants in Yangzonghai Lake were less distributed, with deeper prevailing waters [102]. SOM primarily originates from adjacent rotten vegetation, which impedes its participation in P cycling. It directly combines with P to form residual P, which remains in the sediment. Therefore, the transition of lakes from being grass-dominated to being algae-dominated results in higher P mobility in the sediment [103].

### 4.4. Environmental Significance

This study investigated P-binding forms in the sediments of lakes with varying levels of human activity and trophic ecological types.

Lakes exhibiting high levels of human impacts and abundant algal density, such as Dianchi Lake, have a wide range of exogenous P sources, with algae serving as primary producers in relatively uncomplicated ecosystems [104]. The TP content in the sediments of Dianchi Lake was >2000 mg/kg. Moreover, Dianchi Lake revealed a reduced level of microbial abundance and diversity, thereby facilitating the retention of mobile-P with a content >200 mg/kg. However, elevated levels of mobile-P in sediments pose a high risk of release [43]. For this particular type of lake, it is advisable that, on the basis of external P load control, measures such as sediment dredging or in situ treatment should be taken to control the internal load to effectively manage the internal nutrient load by reducing P levels in the sediment and preventing its potential release from the overlying water.

A grass–algae mixed lake with moderate human activity intensity, such as Erhai Lake, has a low utilization rate of NH_4_Cl-P, and NH_4_Cl-P is easily left in the sediment, reaching an average content of 6.87 mg/kg. The middle part of Erhai Lake lacks aquatic plants because of the deep water depth, causing NH_4_Cl-P in the sediment to be easily released into the water above, leading to a higher chance of eutrophication [105,106]. Submerged vegetation releases O through photosynthesis from the roots as it grows, changing the environment around the roots and causing Fe oxidation. Consequently, Fe oxide can attach to or absorb phosphate, decreasing the re-release of P [107]. Therefore, within the confines of Erhai Lake, which is characterized by a grass–algae ecosystem with uneven vegetation distribution, measures can be taken to restore aquatic vegetation and plant aquatic flora in areas with less vegetation distribution.

Lakes with moderate human activity intensity and mesotrophic algae types, such as Yangzonghai Lake, have less aquatic vegetation distribution, and Res-P is the main P-binding form in the sediments, accounting for 34% of the TP, posing challenges for its release into the overlying waters [108,109]. For lakes with a minimum endogenous pollution load and good water quality, emphasis should be placed on the prevention and management of exogenous pollution, restricting the inflow of nutrients such as N and P, and controlling algal reproduction.

There are significant variations in P morphology within lake sediments, which are influenced by differing intensities of human activity and distinct trophic and ecological types. Therefore, lake management strategies should be tailored to local conditions. To implement targeted scientific management measures, it is essential to conduct comprehensive investigations and measurements of water quality, sediments, and aquatic ecosystems. Moreover, it is crucial to consider the intensity of human activities and the pollution characteristics within watersheds.

## 5. Conclusions

This study investigated P-binding forms in the sediments of three plateau lakes in Yunnan Province—Dianchi Lake, Erhai Lake, and Yangzonghai Lake—which represent eutrophic algae-type, mesotrophic grass–algae mixed-type, and mesotrophic algae-type trophic states, respectively. The findings revealed significant impacts of sediment DOM, human activities, and lake ecosystem characteristics on P retention and mobility, providing new insights into the mechanisms driving internal phosphorus cycling in lakes.

Human activities were found to play a pivotal role in sediment P retention. Correlation analysis revealed strong positive correlations between lake sediment TP, immobile-P, NaOH-rP, and HCl-P with human activity. However, no significant correlation was found between human activity intensity and mobile-P, which is more dynamic and subject to transformation.

Lake ecosystem characteristics affect P mobility in sediments. The mobile-P content in the Dianchi Lake sediment was 40.6% and 278.3% higher than that in Erhai and Yangzonghai, respectively. The ecological shift from grass-dominated to algal-dominated systems increases P mobility because of the activity of microorganisms, algae, and aquatic plants. In addition, shallow water depths enhance P mobility, elevating the risk of internal pollution.

The relationship between sediment DOM and P dynamics is complex. This study showed a significant positive correlation between sediment DOM and mobile-P. On the one hand, DOM may facilitate the accumulation of BD-P through adsorption or hinder the formation of amorphous Fe oxide crystals. On the other hand, DOM acts as a carrier for organophosphorus, impacting microbial processes. This study revealed that the NH_4_Cl-P content in the sediments was positively correlated with the C1 component of a terrestrial humic-like substance and with the C3 component of a protein-like substance. These findings highlight the critical role of DOM composition in shaping sediment P dynamics.

This study provides novel insights into how DOM, human activities, and lake trophic states affect sediment P retention and mobility, with implications for managing internal P loading in eutrophic lakes. The results emphasize the significant role of sediment DOM in modulating phosphorus dynamics, particularly mobile phosphorus forms, while human activities primarily promote the retention of phosphorus in sediments as immobile-P. This study highlights considerable variations in phosphorus forms across lake sediments, driven by human activity and trophic ecological types, underscoring the need for tailored management strategies to control internal phosphorus loading and mitigate eutrophication risks. Future research could explore the interplay between external nutrient inputs and internal P cycling to guide effective lake restoration strategies.

## Data Availability

The data will be made available upon request.

## Supplementary Materials

The following supporting information can be downloaded at: https://www.mdpi.com/article/10.3390/toxics13020120/s1, Section S1: The basic characteristics of Dianchi Lake, Erhai Lake, and Yangzonghai Lake; Section S2: The SOM content and water quality of Dianchi Lake, Erhai Lake, and Yangzonghai Lake; Section S3: Effects of pollution intensity and pollution characteristics on TP content in the sediments of three lakes.

## References

[1] Yan Z.B., Han W.X., Peñuelas J., Sardans J., Elser J.J., Du E.Z., Reich P.B., Fang J.Y. (2016). Phosphorus accumulates faster than nitrogen globally in freshwater ecosystems under anthropogenic impacts. Ecol. Lett..

[2] Diaz R.J., Rosenberg R. (2008). Spreading dead zones and consequences for marine ecosystems. Science.

[3] Zhao J., Gao Q.S., Liu Q.Q., Fu G. (2020). Lake eutrophication recovery trajectories: Some recent findings and challenges ahead. Ecol. Indic..

[4] Ma X.H., Wan H.B., Zhou J., Luo D., Huang T., Yang H., Huang C.C. (2020). Sediment record of polycyclic aromatic hydrocarbons in Dianchi lake, southwest China: Influence of energy structure changes and economic development. Chemosphere.

[5] Pan F., Guo Z.R., Cai Y., Fu Y.Y., Wu J.Y., Wang B., Liu H.T., Gao A.G. (2020). Cyclical patterns and (im) mobilization mechanisms of phosphorus in sediments from a small creek estuary: Evidence from in situ monthly sampling and indoor experiments. Water Res..

[6] Li Q.M., Zhang W., Wang X.X., Zhou Y.Y., Yang H., Ji G.L. (2007). Phosphorus in interstitial water induced by redox potential in sediment of Dianchi Lake, China. Pedosphere.

[7] Hu M.P., Liu Y.M., Zhang Y.F., Shen H., Yao M.Y., Dahlgren R.A., Chen D.J. (2020). Long-term (1980–2015) changes in net anthropogenic phosphorus inputs and riverine phosphorus export in the Yangtze River basin. Water Res..

[8] Yang B., Lan R.Z., Lu D.L., Dan S.F., Kang Z.J., Jiang Q.C., Lan W.L., Zhong Q.P. (2019). Phosphorus biogeochemical cycling in intertidal surface sediments from the Maowei Sea in the northern Beibu Gulf. Reg. Stud. Mar. Sci..

[9] Liang Z., Liu Z., Zhen S., He R. (2015). Phosphorus speciation and effects of environmental factors on release of phosphorus from sediments obtained from Taihu Lake, Tien Lake, and East Lake. Toxicol. Environ. Chem..

[10] Long Z., Ji Z., Pei Y. (2023). Characteristics and distribution of phosphorus in surface sediments of a shallow lake. J. Environ. Sci..

[11] Kong M., Ouyang X.Y., Han T.L., Wang W.Z., Yin H.B., Wang Y. (2024). Combination of lanthanum-modified attapulgite and Vallisneria natans for immobilization of phosphorus in various types of sediments. Chem. Eng. J..

[12] Liu Q., Liu S.L., Zhao H.D., Deng L., Wang C., Zhao Q.H., Dong S.K. (2015). The phosphorus speciations in the sediments up-and down-stream of cascade dams along the middle Lancang River. Chemosphere.

[13] Yin H., Zhang M., Yin P., Li J. (2022). Characterization of internal phosphorus loading in the sediment of a large eutrophic lake (Lake Taihu, China). Water Res..

[14] Duan Z., Tan X., Ali I., Wu X., Cao J., Xu Y., Shi L., Gao W., Ruan Y., Chen C. (2022). Comparison of organic matter (OM) pools in water, suspended particulate matter, and sediments in eutrophic Lake Taihu, China: Implication for dissolved OM tracking, assessment, and management. Sci. Total Environ..

[15] Toming K., Kotta J., Uuemaa E., Sobek S., Kutser T., Tranvik L.J. (2020). Predicting lake dissolved organic carbon at a global scale. Sci. Rep..

[16] Palansooriya K.N., Kim S., Igalavithana A.D., Hashimoto Y., Choi Y.E., Mukhopadhyay R., Sarkar B., Ok Y.S. (2021). Fe (III) loaded chitosan-biochar composite fibers for the removal of phosphate from water. J. Hazard. Mater..

[17] Wang J., Chen Q., Huang S., Wang Z., Li D. (2023). Cyanobacterial organic matter (COM) positive feedback aggravates lake eutrophication by changing the phosphorus release characteristics of sediments. Sci. Total Environ..

[18] Xing X., Chen M., Wu Y., Tang Y., Li C. (2021). The decomposition of macrozoobenthos induces large releases of phosphorus from sediments. Environ. Pollut..

[19] Liu Y., Zhu Z.Q., He X.S., Yang C., Du Y.Q., Huang Y.D., Su P., Wang S., Zheng X.X., Xue Y.J. (2018). Mechanisms of rice straw biochar effects on phosphorus sorption characteristics of acid upland red soils. Chemosphere.

[20] Kurek M., Harir M., Shukle J., Schroth A., Schmitt-Kopplin P., Druschel G. (2021). Seasonal transformations of dissolved organic matter and organic phosphorus in a polymictic basin: Implications for redox-driven eutrophication. Chem. Geol..

[21] Jiang T., Bravo A.G., Skyllberg U., Björn E., Wang D., Yan H., Green N.W. (2018). Influence of dissolved organic matter (DOM) characteristics on dissolved mercury (Hg) species composition in sediment porewater of lakes from southwest China. Water Res..

[22] Ding S., He J., Liu Y., Jiao L.X., Zhao H.C., Cheng Y.X. (2022). The adsorption-release behavior of sediment phosphorus in a typical “grass-algae” coexisting lake and its influence mechanism during the transition sensitive period. Chemosphere.

[23] Bai L.L., Cao C.C., Wang C.L., Wang C.H., Zhang H., Jiang H.L. (2017). Roles of phytoplankton-and macrophyte-derived dissolved organic matter in sulfamethazine adsorption on goethite. Environ. Pollut..

[24] Lin J., Chen X., Liu Y.L., Wang Y.B., Shuai J.X., Chen M.S. (2024). Fe/Mn (oxyhydr) oxides reductive dissolution promoted by cyanobacterial algal bloom-derived dissolved organic matter caused sediment W release during an algal bloom in Taihu Lake. Water Res..

[25] Ni Z., Huang D., Wu Y., Li Y., Zhou C., Wang S. (2023). Intrinsic linkage mechanisms of DOM properties to organic phosphorus in lake sediments: Evidence from coupled molecular weight ultrafiltration and spectral analysis. Chem. Eng. J..

[26] Prüter J., Leipe T., Michalik D., Klysubun W., Leinweber P. (2020). Phosphorus speciation in sediments from the Baltic Sea, evaluated by a multi-method approach. J. Soils Sediments.

[27] Wen S., Liu J., Lu Y., Dai J., Huang X., An S., Jeppesen E., Liu Z., Du Y. (2024). Composition regulates dissolved organic matter adsorption onto iron (oxy)hydroxides and its competition with phosphate: Implications for organic carbon and phosphorus immobilization in lakes. J. Environ. Sci..

[28] Zhao Z., Zhang N., Hu X., Bao L., Xia Y. (2017). Effects of composition and structure of natural organic matter on phosphorus fractions in sediment from Lake Yangzonghai, Yunnan Province. J. Lake Sci..

[29] Chen Q., Ni Z., Wang S., Guo Y., Liu S.R. (2020). Climate change and human activities reduced the burial efficiency of nitrogen and phosphorus in sediment from Dianchi Lake, China. J. Clean. Prod..

[30] Ni Z.K., Wang S.R., Cai J.J., Li H., Jenkins A., Maberly S.C., May L. (2019). The potential role of sediment organic phosphorus in algal growth in a low nutrient lake. Environ. Pollut..

[31] Cui Y., Wen S., Stegen J.C., Hu A., Wang J. (2023). Chemodiversity of riverine dissolved organic matter: Effects of local environments and watershed characteristics. Water Res..

[32] Fan T., Yao X., Sun Z., Sang D., Liu L., Deng H., Zhang Y. (2023). Properties and metal binding behaviors of sediment dissolved organic matter (SDOM) in lakes with different trophic states along the Yangtze River Basin: A comparison and summary. Water Res..

[33] Yang H., Yu J., Xu W., Wu Y., Lei X., Ye J., Geng J., Ding Z. (2023). Long-time series ecological environment quality monitoring and cause analysis in the Dianchi Lake Basin, China. Ecol. Indic..

[34] Luan G., Zhao F., Xia J., Huang Z., Feng S., Song C., Dong P., Zhou X. (2024). Analysis of long-term spatio-temporal changes of plateau urban wetland reveals the response mechanisms of climate and human activities: A case study from Dianchi Lake Basin 1993–2020. Sci. Total Environ..

[35] Zhang Y., Shi T.R., Yu T., Zhang Y. (2013). Sediment Particle Size and the Distribution of Heavy Metals in the Typical Districts of Dianchi Lake. Res. Environ. Sci..

[36] Zhang Y., Shen J., Feng J.M., Li X.Y., Liu H.J., Wang X.Z. (2023). Composition, distribution, and source of organic carbon in surface sediments of Erhai Lake, China. Sci. Total Environ..

[37] Zheng W., Zhang E., Langdon P.G., Wang R. (2024). Systematic loss in biotic heterogeneity but not biodiversity across multiple trophic levels in Erhai lake, China. Sci. Total Environ..

[38] Li H.Y., Zhang H., Chen G., Chang F., Duan L., Wang J., Lu H., Wu H., Hu K. (2017). The Grain Size Distribution Characteristics of Surface Sediments from Plateau Lakes in Yunnan Province and Their Environmental Significances. Acta Sedimentol. Sin..

[39] Yang B., Yang S., Wan X., Hu H., Hu D., Hua M., Liu Y., Pan X. (2020). Temperature models for quantifying groundwater seepage flux applied in a deep lake of a plateau: Yangzonghai Lake, Yunnan, China. Chemosphere.

[40] Xiao H., Mao C., Wang S., Jia Z., Rao W. (2023). Seasonal variation and provenance of organic matter in the surface sediments of the three gorges reservoir: Stable isotope analysis and implications for agricultural management. Sci. Total Environ..

[41] Liu J.J., Zhang Q.Y., Chen M.L., Dai J.R., Gu W.X., Wen S.L., Du Y.X. (2023). Composition of organic matter-iron-phosphorus associations in sediments of algae- and macrophyte-dominated zones in Lake Taihu. Chem. Geol..

[42] Ma J., Yuan Y., Zhou T., Yuan D.X. (2017). Determination of total phosphorus in natural waters with a simple neutral digestion method using sodium persulfate. Limnol. Oceanogr. Methods.

[43] Saha A., Vijaykumar M., Das B., Samanta S., Khan M.F., Kayal T., Jana C., Chowdhury A.R. (2023). Geochemical distribution and forms of phosphorus in the surface sediment of Netravathi-Gurupur estuary, southwestern coast of India. Mar. Pollut. Bull..

[44] He J., Yang Y., Wu X., Zhi G.Q., Zhang Y., Sun X., Jiao L.X., Deng W.M., Zhou H.B., Shao Z. (2022). Responses of dissolved organic matter (DOM) characteristics in eutrophic lake to water diversion from external watershed. Environ. Pollut..

[45] Long Y., Hu X., Jiang J., Hu J., Zhu C., Zhou S. (2021). Phosphorus sorption-desorption behaviors in the sediments cultured with *Hydrilla verticillata* and *Scripus triqueter* as revealed by phosphorus fraction and dissolved organic matter. Chemosphere.

[46] Yang C., Yang P., Geng J., Yin H., Chen K. (2020). Sediment internal nutrient loading in the most polluted area of a shallow eutrophic lake (Lake Chaohu, China) and its contribution to lake eutrophication. Environ. Pollut..

[47] Chen M., Kim S.H., Jung H.J., Hyun J.H., Choi J.H., Lee H.J., Huh I.A., Hur J. (2017). Dynamics of dissolved organic matter in riverine sediments affected by weir impoundments: Production, benthic flux, and environmental implications. Water Res..

[48] Panettieri M., Guigue J., Prévost-Bouré N.C., Thévenot M., Lévêque J., Guillou L., Maron P.A., Santoni A.L., Ranjard L., Mounier S. (2020). Grassland-cropland rotation cycles in crop-livestock farming systems regulate priming effect potential in soils through modulation of microbial communities, composition of soil organic matter and abiotic soil properties. Agric. Ecosyst. Environ..

[49] Borisover M., Laor Y., Parparov A., Bukhanovsky N., Lado M. (2009). Spatial and seasonal patterns of fluorescent organic matter in Lake Kinneret (Sea of Galilee) and its catchment basin. Water Res..

[50] Yamashita Y., Cory R.M., Nishioka J., Kuma K., Tanoue E., Jaffé R. (2010). Fluorescence characteristics of dissolved organic matter in the deep waters of the Okhotsk Sea and the northwestern North Pacific Ocean. Deep. Sea Res. Part II Top. Stud. Oceanogr..

[51] Rapin A., Grybos M., Rabiet M., Mourier B., Deluchat V. (2019). Phosphorus mobility in dam reservoir affected by redox oscillations: An experimental study. J. Environ. Sci..

[52] Ding S., Liu Y., Dan S.F., Jiao L.X. (2021). Historical changes of sedimentary P-binding forms and their ecological driving mechanism in a typical “grass-algae” eutrophic lake. Water Res..

[53] Wang J.H., Yang C., Dao G.H., Du J.S., Han Y.P., Wu G.X., Wu Q.Y., Hu H.Y. (2019). Meteorological factors and water quality changes of Plateau Lake Dianchi in China (1990–2015) and their joint influences on cyanobacterial blooms. Sci. Total Environ..

[54] Ren Z., He J., Cheng Q., Ding S., Liu W., Duan P., Jiao L.X. (2022). Climate change prior to human activity reduces the immobility of phosphorus in eutrophic alpine lake. J. Clean. Prod..

[55] Shi M., Li J., Zhou Q., Wang G., Zhang W., Zhang Z., Gao Y., Yan S. (2020). Interactions between elevated CO_2_ levels and floating aquatic plants on the alteration of bacterial function in carbon assimilation and decomposition in eutrophic waters. Water Res..

[56] Zhang Y., Zhang T., Huang D., Shao D., Luo H. (2022). Geochemical and paleontological evidence of early Cambrian dynamic ocean oxygenation and its implications for organic matter accumulation in mudrocks at the Three Gorges area, South China. Mar. Pet. Geol..

[57] Pontoni L., LaVecchia C., Boguta P., Sirakov M., D’Aniello E., Fabbricino M., Locascio A. (2022). Natural organic matter controls metal speciation and toxicity for marine organisms: A review. Environ. Chem. Lett..

[58] Zhu B., Cheng W. (2013). Impacts of drying–wetting cycles on rhizosphere respiration and soil organic matter decomposition. Soil. Biol. Biochem..

[59] Zhu C., Wang Z.H., Xue B., Yu P.S., Pan J.M., Wagner T., Pancost R.D. (2011). Characterizing the depositional settings for sedimentary organic matter distributions in the Lower Yangtze River-East China Sea Shelf System. Estuar. Coast. Shelf Sci..

[60] Ding X., Guo X., Gao H., Gao J., Shi J., Yu X., Wu Z. (2021). Seasonal variations of nutrient concentrations and their ratios in the central Bohai Sea. Sci. Total Environ..

[61] Ren H., Fan T., Yao X., Ma F., Liu L., Ming J., Wang S., Zhang Y., Deng H. (2022). Investigation of the variations in dissolved organic matter properties and complexations with two typical heavy metals under the influence of biodegradation: A survey of an entire lake. Sci. Total Environ..

[62] Wang H.B., Liu X.P., Jin B.J., Shu Y.C., Sun C.L., Zhu Y.G., Lin X.Y. (2024). High-molecular-weight dissolved organic matter enhanced phosphorus availability in paddy soils: Evidence from field and microcosm experiments. Soil. Tillage Res..

[63] Zhang T., Chen L., Liu X., Shang L., Liu Y., Wang C., Zhao S., Chen G. (2020). Spatial pattern and influencing factors of phytoplankton in lakes of central and southern Yunnan in summer. Chin. J. Ecol..

[64] Wang S., Jin X., Zhao H., Zhou X., Wu F. (2007). Effect of organic matter on the sorption of dissolved organic and inorganic phosphorus in lake sediments. Colloids Surf. A.

[65] Wang S., Yi W., Yang S., Jin X., Wang G., Wu F. (2011). Effects of light fraction organic matter removal on phosphate adsorption by lake sediments. Appl. Geochem..

[66] Wang S.R., Jin X.C., Zhao H.C., Zhou X.N., Wu F.C. (2008). Effects of organic matter on phosphorus release kinetics in different trophic lake sediments and application of transition state theory. J. Environ. Manag..

[67] Du S.T., Shentu J.L., Luo B.F., Shamsi I.H., Lin X.Y., Zhang Y.S., Jin C.W. (2011). Facilitation of phosphorus adsorption onto sediment by aquatic plant debris. J. Hazard. Mater..

[68] Jin J.Q., Xu J., Mo Y., Tang H., Wei T., Wang Y.G., Li L. (2020). Response of sediments and phosphorus to catchment characteristics and human activities under different rainfall patterns with Bayesian Networks. J. Hydrol..

[69] Martinez-Escobar D.F., Mallela J. (2019). Assessing the impacts of phosphate mining on coral reef communities and reef development. Sci. Total Environ..

[70] Rashmi K., Karthika T., Roy K., Shinoji A., Kumawat S., Kala R.P. (2022). Agrochemicals in Soil and Environment: Impacts and Remediation.

[71] Tang R., Bai J., Zhang L., Wang Y., Liu H., Xia J. (2024). Advances and hotspots of phosphorus form analysis of sediments based on different extraction methods using CiteSpace. Ecohydrol. Hydrobiol..

[72] Wen S., Zhong J., Li X., Liu C., Yin H., Li D., Ding S., Fan C. (2020). Does external phosphorus loading diminish the effect of sediment dredging on internal phosphorus loading? An in-situ simulation study. J. Hazard. Mater..

[73] Ye H., Yuan X., Han L., Yin H., Jin J. (2017). Comparison of phosphorus fraction distribution and influencing factors of suspended and surface sediments in the Tiaoxi watershed, China. Water Sci. Technol..

[74] Dittrich M., Chesnyuk A., Gudimov A., McCulloc J., Quazi S., Young J., Winter J., Stainsby E., Arhonditsis G. (2013). Phosphorus retention in a mesotrophic lake under transient loading conditions: Insights from a sediment phosphorus binding form study. Water Res..

[75] Wang S., Zhang M., Li B., Xing D., Wang X., Wei C., Jia Y. (2012). Comparison of mercury speciation and distribution in the water column and sediments between the algal type zone and the macrophytic type zone in a hypereutrophic lake (Dianchi Lake) in Southwestern China. Sci. Total Environ..

[76] Zhang Z., Jiang L., Chen M., Li J., Zhang L., Zhang J., Liao N. (2022). Release and transformation of phosphorus in sediment following seasonal freezing-thawing cycles. J. Contam. Hydrol..

[77] Wang J., Mu X., Chen S., Liu W., Wang Z., Dong Z. (2021). Dynamic evaluation of water resources carrying capacity of the Dianchi Lake Basin in 2005–2015, based on DSPERM framework model and simulated annealing-projection pursuit model. Reg. Sustain..

[78] Wang S., Wang J., Li M., Du F., Yang Y., Lassoie J.P., Hassan M.Z. (2013). Six decades of changes in vascular hydrophyte and fish species in three plateau lakes in Yunnan, China. Biodivers. Conserv..

[79] Yan K., Yuan Z., Goldberg S., Gao W., Ostermann A., Xu J., Zhang F., Elser J. (2019). Phosphorus mitigation remains critical in water protection: A review and meta-analysis from one of China’s most eutrophicated lakes. Sci. Total Environ..

[80] Han X., Feng L., Chen X., Yesou H. (2014). MERIS observations of chlorophyll-a dynamics in Erhai Lake between 2003 and 2009. Int. J. Remote Sens..

[81] Xie G., Nu W., Yang S. (2007). Land use and water quality changes in Yangzonghai Lake, Songhuaba Reservoir and Yunlong Reservoir basins. Water Resour. Prot..

[82] Rauch J.N. (2010). Global spatial indexing of the human impact on Al, Cu, Fe, and Zn mobilization. Environ. Sci. Technol..

[83] Lin J., Sun Q., Ding S., Wang D., Wang Y., Chen M., Shi L., Fan X., Tsang D.C. (2017). Mobile phosphorus stratification in sediments by aluminum immobilization. Chemosphere.

[84] Zhou Z., Wang Y., Yang H., Liu A., Wu S., Teng H., Niu X.Y. (2021). Sedimentary record of nutrients and sources of organic matter in the Shuanglong reservoir, Dianchi watershed, China. Environ. Sci. Pollut. Res..

[85] Klamt A.M., Qian F., Hu K., Wang J., Huang L., Li R., Chen G. (2021). The rise and fall of primary producers and consumers in a multiply-stressed shallow lake (Lake Qilu, China) over the last 200 years. Ecol. Indic..

[86] He L., Chen G., Wang X., Shen J., Zhang H., Lin Y., Shen Y., Lang F., Gong C.L. (2024). Pollution Characteristics and Risk Assessment of Heavy Metals in the Sediments of the Inflow Rivers of Dianchi Lake, China. Toxics.

[87] Zhu R., Wang H., Chen J., Shen H., Deng X.W. (2018). Use the predictive models to explore the key factors affecting phytoplankton succession in Lake Erhai, China. Environ. Sci. Pollut. Res..

[88] Pan Y.H., Wang H.B., Gu Z.P., Xiong G.H., Yi F. (2010). Accumulation and translocation of heavy metals by macrophytes. Acta Ecol. Sin..

[89] Zhu Y., Wu F., He Z., Giesy J.P., Feng W., Mu Y., Feng C., Zhao X., Liao H., Tang Z. (2015). Influence of natural organic matter on the bioavailability and preservation of organic phosphorus in lake sediments. Chem. Geol..

[90] Takahashi T., Dahlgren R.A. (2016). Nature, properties and function of aluminum–humus complexes in volcanic soils. Geoderma.

[91] Bao Y., Huang T., Ning C.W., Sun T.T., Tao P.L., Wang J., Sun Q.Y. (2023). Changes of DOM and its correlation with internal nutrient release during cyanobacterial growth and decline in Lake Chaohu, China. J. Environ. Sci..

[92] Wu W., Yuan L., Li E., Liu W., Li W. (2007). Effects of Aquatic Macrophyte on Phosphorus in Lake Sediment. Hubei Agric. Sci..

[93] Wang J., Gao M., Yang Y., Lu S., Wang G., Qian X.Q. (2022). Interactions of vallisneria natans and iron-oxidizing bacteria enhance iron-bound phosphorus formation in eutrophic Lake sediments. Microorganisms.

[94] Cheng Y.X., Jiao L.X., Cheng Q.L., He J., Zhang Y., Ding S. (2023). The evolution of a typical plateau lake from macrophyte to algae leads to the imbalance of nutrient retention. Water Res..

[95] Yin H., Yin P., Yang Z. (2023). Seasonal sediment phosphorus release across sediment-water interface and its potential role in supporting algal blooms in a large shallow eutrophic Lake (Lake Taihu, China). Sci. Total Environ..

[96] Zhang Y., Zhang C., Qin J., Chen Z., Chen Y., Li J., Wang X. (2023). Introducing La (OH)_3_ nanoparticles into attapulgite for the control of sediments internal phosphorus release: Effectiveness, mechanisms, and microbial community response. J. Environ. Chem. Eng..

[97] Wang S.R., Zhang L., Ni L.Y., Zhao H.C., Jiao L.X., Yang S.W., Guo L.G., Shen J.Z. (2015). Ecological degeneration of the Erhai Lake and prevention measures. Environ. Earth Sci..

[98] Yang S., Yu X.L., Gao D., Zhang K., Wu J., Zhang X., Cui Y. (2023). Health Assessment on Lake Erhai Using Benthic Index of Biotic Integrity. Environ. Sci. Technol..

[99] Liu C., Du Y., Zhong J., Zhang L., Huang W., Han C., Chen K., Gu X. (2022). From macrophyte to algae: Differentiated dominant processes for internal phosphorus release induced by suspended particulate matter deposition. Water Res..

[100] Liu S., Wang J., Lin C., He M., Liu X. (2013). Geochemical baseline level and function and contamination of phosphorus in Liao River Watershed sediments of China. J. Environ. Manag..

[101] Wilson T.A., Amirbahman A., Norton S.A., Voytek M.A. (2010). A record of phosphorus dynamics in oligotrophic lake sediment. J. Paleolimnol..

[102] Zheng X., Chen L., Qiu F., Zhang T., Zhang Z., Shang L., Bai N., Chen X., Zhao S., Kong L. (2024). Spatio-temporal distribution and driving factors of phytoplankton biomass in Lake Yangzong under the background of arsenic pollution treatment. J. Lake Sci..

[103] Zhang J., Zuo A., Salimova L., Aijun L., Ling L. (2020). Phytoplankton distribution characteristics and its relationship with bacterioplankton in Dianchi Lake. Environ. Sci. Pollut. Res..

[104] Li G., Li L., Pan M., Xie Z., Li Z., Xiao B., Liu G., Chen J., Song L. (2014). The degradation cause and pattern characteristics of Lake Dianchi ecosystem and new restoration strategy of ecoregion and step-by-step implementation. J. Lake Sci..

[105] Chu Z., Ye B., Tian G., Pang Y., Hu X. (2014). Spatial distribution characteristics and estimation of submerged plant biomass in Lake Erhai. Res. Environ. Sci..

[106] Tian Y., Chen X., Lv C., Shan H., Chou Q., Lv X., Zhang X., Ni L., Cao T. (2023). Diversity and distribution status of aquatic plants in the lakeshore zone of Lake Erhai. J. Lake Sci..

[107] Xu D., Yan P., Liu Z., Zhang M., Yan W., Liu Y., Wu Z., Zhang Y. (2021). Spatial distribution of phosphorus forms and the release risk of sediments phosphorus in West Lake, Hangzhou, China. Ecol. Eng..

[108] Tao J., Chen G., Chen X., Chen L., Huang L., Liu Y., Shi H., Hu K., Wang J., Kang W. (2016). Long-term pattern of diatom community responses to water pollution and hydrological regulation in Yangzong Lake. Geogr. Res..

[109] Zhang H., Li Q., Zhang X., Chen W., Ni J., Yang L., Wei R. (2020). Insight into the mechanism of low molecular weight organic acids-mediated release of phosphorus and potassium from biochars. Sci. Total Environ..

