# Supplementary material for: Influencing Factors of Phosphorus Mobility and Retention in the Sediment of Three Typical Plateau Lakes
Source: Toxics. 2025 Feb 3;13(2):120. doi: 10.3390/toxics13020120 (PMC11860908; doi:10.3390/toxics13020120)
Supplement: Supplementary file 1 [file toxics-13-00120-s001.zip › toxics-3410017-supplementary.pdf]

Supplementary Material for

Influencing Factors of Phosphorus Mobility and Retention in the Sediment of Three Typical Plateau Lakes

Section S1

The basic characteristics of Dianchi Lake, Erhai Lake, and Yangzonghai Lake are shown in Table S1.

Despite being plateau lakes, Dianchi Lake, Erhai Lake, and Yangzonghai Lake exhibit unique differences in terms of socio-economic development, natural conditions, and ecological characteristics. Table S1 displays the contrasting features of Dianchi Lake, Erhai Lake, and Yangzonghai Lake. Dianchi Lake has the largest basin area, lake area, unit area, and population density and is followed by Erhai Lake and Yangzonghai Lake. The hydraulic retention time of Yangzonghai Lake is 11.57 years, notably surpassing that of Dianchi Lake by 4.9 times and Erhai Lake by 4.2 times. Interestingly, Dianchi Lake has the same hydraulic retention time as Erhai Lake. In addition, Yangzonghai Lake has an average water depth of 20.13 m, which is much deeper than Dianchi Lake (4.4 m) and Erhai Lake (10.5 m) [1-3]. Dianchi Lake falls into the category of moderately eutrophic among the various trophic types of lakes, whereas Erhai Lake and Yangzonghai Lake are classified as mesotrophic. The level of eutrophication of Dianchi Lake surpasses Erhai Lake and Yangzonghai Lake, resulting in lower transparency and DO. In terms of aquatic ecological characteristics, Dianchi Lake harbors up to 138 species of phytoplankton, with cyanophyta being the most abundant and accounting for 87% of total species, while chlorophyta and bacillariophyta account for similar proportions [4-5]. On the other hand, Erhai Lake hosts 74 phytoplankton species, with chlorophyta and bacillariophyta as the dominant, and the proportion of cyanophyta is substantially lesser than in Dianchi Lake [6-7]. It should be noted that Yangzonghai Lake harbors at least 33 phytoplankton species with equal proportions of chlorophyta, cyanophyta, and bacillariophyta as formidable species [8-9]. This reveals a sizeable risk of algal bloom in Dianchi Lake compared to Erhai Lake and Yangzonghai Lake. Moreover, Erhai Lake features a richer presence of benthonic animals and aquatic plants in comparison to Dianchi Lake and Yangzonghai Lake, showcasing the favorable biodiversity of Erhai Lake [10-12].

Table S1. The basic characteristics of typical plateau lakes.

Indicators	Dianchi Lake	Erhai Lake	Yangzonghai Lake
Basin area of lake (km^2)	2920	2565	286
Lake area (km^2)	297.9	249.8	31.3
Unit area of GDP (ten thousand yuan)	11149.83	1301.28	452.48
Population density ($person/km^2$)	1868	325	212
Urbanization rate (%)	70	63	65.2
Urban land area ratio (%)	25.17	7.94	5.52
Hydraulic retention time (a)	2.34	2.75	11.57
Average water depth (m)	4.4	10.5	20.13
Nutrient type of lake	Moderately eutrophic	Mesotrophic	Mesotrophic

Transparency (m)	0.55	1.6	2.2
Dissolved oxygen level	5.85	7.03	7.93
pH value	8.28	8.72	8.48
	Phytoplankton		
Species	138	74	33
Chlorophyta (%)	7	37.2	27.27
Cyanophyta (%)	87	14	27.27
Bacillario-phyta (%)	6	39.5	27.27
	Zoobenthos		
Species	23	56	20
Oligochaeta (g/m^2)	0.54	0.2	0.44
Chironomidae (g/m^2)	0.65	0.7	0.08
	Aquatic plants		
Species	17	206	17
Biomass（$\times 10^4$t）	5.95	16	/

Section S2

The SOM content and water quality of Dianchi Lake, Erhai Lake, and Yangzonghai Lake are shown in Table S2.

Table S2. The SOM content and water quality of the three lakes

Lake	Flow direction	Sampling points	Lake area	Water depth/m	Sediment OM(%)	Sediment DOM(mg/kg)	Overlying water quality (mg/L)							Porewater SRP (mg/L)
							COD	TN	TP	NH_3-N	Chla	TLI	SRP	
Dianchi Lake	North to south	C1	Caohai	3	45.36	3466.07	12	2.6	0.06	0.04	0.06	58.67	0.036	0.33
		W1	North Waihai	2.9	3.78	1074.8	35.5	1.74	0.068	0.2	0.11	62.46	0.04	0.34
		W2	Middle Waihai	6.1	15.29	541.32	31.5	1.32	0.064	0.32	0.12	61.89	0.014	0.07
		W3	South Waihai	5.6	13.55	1480.32	32	1.48	0.066	0.15	0.113	63.31	0.013	0.13
Erhai Lake	North to south	E1	North	7.49	4.87	1953.64	13	0.46	0.02	0.03	0.005	36.97	0.015	0.007
		E2	Middle	19.59	3.97	990.5	14	0.37	0.017	0.02	0.014	40.18	0.014	0.04
		E3	South	4.93	5.54	1041.47	12.5	0.48	0.02	0.04	0.01	39.44	0.016	0.0354
Yangzonghai Lake	South to nouth	Y1	North	18.1	7.00	933.76	12	0.83	0.02	0.03	0.008	40.4	0.013	0.0304
		Y2	Middle	22.6	6.47	1121.58	9	0.61	0.02	0.03	0.011	41.26	0.015	0.0495
		Y3	South	21.3	5.21	980.27	12	0.82	0.02	0.03	0.007	40.33	0.016	0.0279

Section S3

Among the three lakes in this study, Dianchi Lake has the highest sediment TP content, which is affected by pollution intensity and pollution source characteristics.

The first factor to comprehend is the influence of pollution intensity. Dianchi Lake basin exhibits higher population density and urbanization than the Erhai Lake and Yangzonghai Lake basins. This signifies that the Dianchi Lake basin is more disturbed by human activities and has a relatively higher P pollution load. For instance, the population density of the Dianchi Lake basin is 1,868 persons/km^2, with a GDP per unit area of RMB 111,498,300 yuan/km^2, an urbanization rate of 70%, and the urban land area constituting 25.17% [13]. In comparison, the population density of the Erhai Lake basin is 325 persons/km^2, with a GDP per unit area of RMB 13,012,800 yuan/km^2, an urbanization rate of 63%, and the urban land area accounting for 7.94%. Similarly, the population density of the Yangzonghai Lake basin is 212 persons/km^2, with a GDP per unit area of RMB 4,524,800 yuan/km^2, an urbanization rate of 65.2%, and an urban land area constituting 5.52%.

The second factor to consider is the characteristics of pollution sources. Dianchi Lake is located downstream of the main urban area of Kunming, which is chiefly impacted by urban domestic pollution discharge. Meanwhile, Erhai Lake and Yangzonghai Lake are extensively affected by agricultural and rural non-point source pollution. It is noteworthy that Caohai Lake and the northern zone of Waihai Lake are adjacent to the central urban area of Kunming, which is characterized by a high population density and greatly influenced by anthropogenic activities. Additonally, the central part of Waihai Lake lies within the Kunming urban area, with a sparse population density compared to the northern zone, resulting in lesser disturbance to sediments. The southern zone of Waihai Lake borders Jinning District, which is susceptible to industrial and agricultural non-point source pollution due to the phosphate-rich and agricultural nature of the area [14]. The rivers, including Miju River, Luoshi River, and Yong'an River, flow into Erhai Lake and are distributed in the northern zone. Miju River passes through Eryuan County, which is dominated by agriculture and prone to serious agricultural non-point source pollution. In addition, the central part of Erhai Lake is close to Wase town and houses a relatively lower population density compared to the northern and southern zones [15]. In the southern zone of Erhai Lake the Boluo River is the main tributary, with limited water flow, located near Xiaguan District in Dali Prefecture. This region has a dense population and is adjacent to various building material factories. The land use patterns in the Yangzonghai basin mainly include farmland, forest, and grassland [16]. The northern zone of Yangzonghai Lake neighbors Tangchi Town, with concentrated human activities and farmland distribution. The Baiyi River in the northern zone serves as an entry point for domestic sewage into the lake, surrounded by industrial and mining enterprises such as power plants, coal mines, and tourism services. There are aluminum factories and coal ash storage yards near the center of Yangzonghai, with a lower population density compared to that in the northern and southern zones. The southern zone of Yangzonghai is adjacent to Yangzong Town, with relatively intense human activities and farmland, phosphate fertilizer plants, and refractory plants distributed around it. Domestic sewage enters the lake through the Yangzong River in the southern zone.

References

1. Zhu, R.; Wang, H.; Chen, J.; Shen, H.; Deng, X.W. Use the predictive models to explore the key factors affecting phytoplankton succession in Lake Erhai, China. *Environ Sci Pollut Res.* **2018**, 25, 1283-1293.
2. Pan, Y.H.; Wang, H.B.; Gu, Z.P.; Xiong,G.H.; Yi, F. Accumulation and translocation of heavy metals by macrophytes. *Acta Ecolo Sin.* **2010**, 30, 6430-6441.

3. He, L.; Chen, G.; Wang, X.; Shen, J.; Zhang, H.; Lin, Y.; Shen, Y.; Lang, F.; Gong, C.L. Pollution Characteristics and Risk Assessment of Heavy Metals in the Sediments of the Inflow Rivers of Dianchi Lake, China. *Toxics.* **2024**, 12, 322.
4. He,J.; Yang, Y.; Wu, X.; Zhi, G.Q.; Zhang, Y.; Sun, X.; Jiao, L.X.; Deng, W.M.; Zhou, H.B.; Shao, Z.; Zhu, Q.F. Responses of dissolved organic matter (DOM) characteristics in eutrophic lake to water diversion from external watershed. *Environ Pollut.* **2022**, 312, 119992.
5. Li, G.; Li, L.; Pan, M.; Xie, Z.; Li, Z.; Xiao, B.; Liu, G.; Chen, J.; Song, L. The degradation cause and pattern characteristics of Lake Dianchi ecosystem and new restoration strategy of ecoregion and step-by-step implementation. *J Lake Sci.* **2014**, 26, 485-496.
6. Chu, Z.; Ye, B.; Tian, G.; Pang, Y.; Hu, X. Spatial distribution characteristics and estimation of submerged plant biomass in Lake Erhai. *Res Environ Sci.* **2014**, 27, 1-5.
7. Tian, Y.; Chen, X.; Lv, C.; Shan, H.; Chou, Q.; Lv, X.; Zhang, X.; Ni, L.; Cao, T. Diversity and distribution status of aquatic plants in the lakeshore zone of Lake Erhai. *J Lake Sci.* **2023**, 35, 941-949.
8. Tao, J.; Chen, G.; Chen, X.; Chen, L.; Huang, L.; Liu, Y.; Shi, H.; Hu, K.; Wang, J.; Kang, W. Long-term pattern of diatom community responses to water pollution and hydrological regulation in Yangzong Lake. *Geogr Res.* **2016**, 35, 1899-1911.
9. Zhang, H.; Li, Q.; Zhang, X.; Chen, W.; Ni, J.; Yang, L.; Wei, R. Insight into the mechanism of low molecular weight organic acids-mediated release of phosphorus and potassium from biochars. *Sci Total Environ.* **2020**, 742, 140416.
10. Wang, S.R. Zhang, L.; Ni, L.Y.; Zhao, H.C.; Jiao, L.X.; Yang, S.W.; Guo, L.G.; Shen J.Z. Ecological degeneration of the Erhai Lake and prevention measures. *Environ Earth Sci.* **2015**, 74, 3839-3847.
11. Yang, S.; yu, X.L.; Gao, D.; Zhang, K.; Wu, J.; Zhang, X.; Cui, Y. Health Assessment on Lake Erhai Using Benthic Index of Biotic Integrity. *Environ Sci Technol.* **2023**, 46, 181-188.
12. Zheng, X.; Chen, L.; Qiu, F.; Zhang, T.; Zhang, Z.; Shang, L.; Bai, N.; Chen,X.; Zhao, S.; Kong, L.; Chen, G. Spatio-temporal distribution and driving factors of phytoplankton biomass in Lake Yangzong under the background of arsenic pollution treatment. *J Lake Sci.* **2024**, 36, 34-42.
13. Wang, J.; Mu, X.; Chen, S.; Liu, W.; Wang, Z.; Dong, Z. Dynamic evaluation of water resources carrying capacity of the Dianchi Lake Basin in 2005–2015, based on DSPERM framework model and simulated annealing-projection pursuit model. *Reg Sustainability.* **2021**, 2, 189-201.
14. Wang, S.; Zhang, M.; Li, B.; Xing, D.; Wang, X.; Wei, C.; Jia, Y. Comparison of mercury speciation and distribution in the water column and sediments between the algal type zone and the macrophytic type zone in a hypereutrophic lake (Dianchi Lake) in Southwestern China. *Sci Total Environ.* **2012,** 417, 204-213.
15. Han, X.; Feng, L.; Chen, X.; Yesou, H. MERIS observations of chlorophyll-a dynamics in Erhai Lake between 2003 and 2009. *Int J Remote Sens.* **2014**, 35, 8309-8322.
16. Xie, G.; Nu, W.; Yang, S. Land use and water quality changes in Yangzonghai Lake, Songhuaba Reservoir and Yunlong Reservoir basins. *Water Resour Prot.* **2007**, 5, 15-17.
